# Maximization of Livestock Anthrax Vaccination Coverage in Bangladesh: An Alternative Approach

**DOI:** 10.3390/vaccines8030435

**Published:** 2020-08-04

**Authors:** M. Shahjahan A. Sarker, Mohamed E. El Zowalaty, M. Ahosanul Haque Shahid, M. Asaduzzaman Sarker, M. Bahanur Rahman, Josef D. Järhult, K. H. M. Nazmul Hussain Nazir

**Affiliations:** 1 Department of Microbiology and Hygiene, Faculty of Veterinary Science, Bangladesh Agricultural University, Mymensingh 2202, Bangladesh; shahjahansarker1981@gmail.com (M.S.A.S.); shahid41192@bau.edu.bd (M.A.H.S.); bahanurr@bau.edu.bd (M.B.R.); 2 Zoonosis Science Center, Department of Medical Biochemistry and Microbiology, Uppsala University, SE 75123 Uppsala, Sweden; 3 Department of Clinical Sciences, College of Medicine, University of Sharjah, Sharjah 27272, UAE; 4 Department of Agricultural Extension Education, Bangladesh Agricultural University, Mymensingh 2202, Bangladesh; masarker@bau.edu.bd; 5 Zoonosis Science Center, Department of Medical Sciences, Uppsala University, SE 75185 Uppsala, Sweden; josef.jarhult@medsci.uu.se

**Keywords:** *Bacillus anthracis*, Anthrax, vaccine, Livestock, vaccination, challenges, zoonosis, Bangladesh

## Abstract

Low vaccination coverage of livestock is one of the major challenges to control anthrax in Bangladesh. This study was conducted to assess an alternate approach to maximize vaccination coverage. The method included traditional vaccination campaigns, livestock census, interviews, focus group discussions of cattle farmers, vaccination and livestock personnel, and validation workshops. It was observed that a mass vaccination program covered only 44% of the cattle population. It was found that 54.1% of the respondents did not bring their cattle to mass vaccination programs due to the difficulties of handling cattle and that there was no male member in the household. Only 12.5% of respondents acknowledged that they were not aware of the vaccine, and 3% of the respondents claimed that they ignored vaccination due to cost. All of the respondents from livestock personnel agreed that manpower was not enough to cover the total area. Further, 20% of vaccinators mentioned that they did not get enough vaccines. For an effective vaccination program, 58.33% of respondents recommended door-to-door service, and 54.16% of respondents suggested to arrange regular vaccination campaigns in six-month intervals. Thus, regular campaigns with door-to-door vaccination services are suggested to control anthrax outbreaks in Bangladesh.

## 1. Introduction

Bangladesh is one of the most densely populated countries in the world. It also has the highest density of livestock such as cattle, goat, sheep, and buffaloes as compared to other countries. Rural households mostly rear livestock as a part of their daily lives, and it plays an important role in their livelihood. The contribution of livestock is multidimensional. It is not only a source of quality protein, but also its manure is the best organic fertilizer for land, bone is an important source of minerals, while skin and hides are the primary raw material of leather industries. Many sectors and professions like veterinarians, butchers, livestock traders, artificial inseminators, medicine suppliers and marketing networkers, livestock feed industries, and dairy and meat food processing industries depend on livestock. It also serves as a farmer’s bank that helps locals to deal with an emergency. In the rural community, livestock owners are mostly landless, marginal, and have a small amount of cultivable land. Livestock plays an important economic and socio-cultural role not only in rural households but also in the national economy. About 20% of the human population is directly, and about 50% is somehow dependent on this sector [1]. The contribution of the livestock sector in the national gross domestic product (GDP) is 1.54% [2,3,4]. There are many challenges like poor productivity, lack of pastureland, traditional husbandry knowledge, unstable market situation, emerging diseases, and insufficiency of veterinary service that are considered as major threats for livestock production. Unfortunately, some infectious diseases strike regularly, causing the death of animals and loss in the production, hampering incomes of families and food security, and livelihood of rural communities [2,3]. Foot and mouth disease (FMD), hemorrhagic septicemia (HS), anthrax, rabies, and peste des petits ruminants (also known as Pseudorinderpest, Goat Plague, Pest of Small Ruminants) are the most common infectious diseases affecting livestock in Bangladesh [4]. These diseases cause thousands to millions of animal deaths, and some of them have zoonotic potential, i.e., can transmit from animals to humans. Anthrax (caused by *Bacillus anthracis*, a soil-borne spore-forming Gram-positive bacterium) is endemic in South Asian countries, including Bangladesh. The anthrax spore is viable for several decades in soil under environmental conditions, and it is resistant to heat and chemical disinfectants. Herbivore animals, mostly cattle, become infected when a low number (5 × 10^8^) of spores enter the body through contaminated feed and water [5]. After entering, the spores germinate and multiply inside the host body within 3–7 days [5]. In severe cases, massive terminal bacteremia and toxin production lead to death. Vegetative bacilli are shed in blood and discharge form the carcass or infected animals. Humans become infected through contact with infected animals and animal products. Anthrax is also considered an occupational disease affecting people who raise livestock, veterinarians, people who work in leather industries, and those involved in animal slaughter, processing, and the selling of meat and animal products. Humans become infected when the spores contaminate a cut or scrape of skin, eat contaminated food, or inhale spores [6,7,8,9]. In humans, there are three forms of infection, namely cutaneous, gastrointestinal, and inhalation. Anthrax was reported periodically in Bangladesh, however in the last few years, there were repeated outbreaks of anthrax in both humans and animals, causing hundreds to thousands of livestock deaths [9]. In humans, most of the cases were cutaneous, and in 2011 two human fatalities occurred in two different locations [10]. Both patients were male, aged 70 years and 40 years, respectively, and had a cutaneous infection along with gastrointestinal symptoms [10]. Although very few humans have died from anthrax, the outbreak has created a panic situation among people of Bangladesh. Many people avoided eating beef, and the livestock market fell sharply. As a result, a negative impact was seen in the livestock sector and rural economy [10,11,12,13].

Anthrax was reported to be hyperendemic in many countries of the world. The World Organization for Animal Health (OIE), World Health Organization (WHO), and the Food and Agricultural Organization of United Nations (FAO) jointly developed anthrax control guidelines. Through implementing effective prevention measures, there has been a progressive reduction of anthrax cases over the past three decades [14]. Now many European countries, North America, and Australia have controlled anthrax, and the disease is now absent or only sporadic in those countries. In Africa, Zambia was identified as a model country of the anthrax control program. Regular vaccinations, increased public awareness, and proper quarantine were considered as a major strategy to control anthrax all over the world [15,16,17].

Many exploratory studies were performed in Bangladesh to find out the reasons of repeated outbreaks of anthrax, and many of them have suggested measures to control the disease. Most of the studies agreed that limited vaccination coverage was one of the most important contributing factors, and studies recommended that regular effective vaccination may reduce anthrax infection [18,19,20,21]. Anthrax vaccine for livestock is available in Bangladesh and manufactured by the Livestock Research Institute (LRI, Dhaka, Bangladesh), a government-regulated and -owned institute under the Ministry of Fisheries and Livestock. The Australia-origin Sterne F-34 vaccine strain of *B. anthracis* is used as a master seed of vaccine production. One milliliter (1 × 10^7^ spores approximately) of the vaccine is used as subcutaneous injection for cattle and 0.5 mL for small ruminants such as sheep and goats. Despite some technical difficulties associated with the Sterne F-34 non-capsulated attenuated spore vaccine, including local swelling, painful irritation during injecting, elevated body temperature, and reduced milk yield, it is quite effective to protect the animal for a year [19]. In field trials, it was reported that after proper vaccination with this vaccine, protective serum antibody levels developed within 21 days and remained protective for up to one year [4,18,19,20]. Human anthrax mostly originates from livestock and thus control of anthrax in animals is the best strategy to control disease in humans. Considering herd immunity, most researchers recommend that for effective control, at least eighty percent of animals should be effectively vaccinated [21,22,23].

Considering the overall situation in Bangladesh, the present study was conducted to assess the status of mass anthrax vaccination coverage of livestock in the rural community. Moreover, an attempt was made to explore the major challenges to achieve a successful vaccination program and explore the possible strategies for a successful mass vaccination program to establish an anthrax-free model area in Bangladesh.

## 2. Materials and Methods

### 2.1. Ethics

The study was approved by the Animal Welfare and Experimentation Ethics Committee, Bangladesh Agricultural University [Reference number AWEEC/BAU/2018(21)].

### 2.2. Study Sites

**Figure 1:**
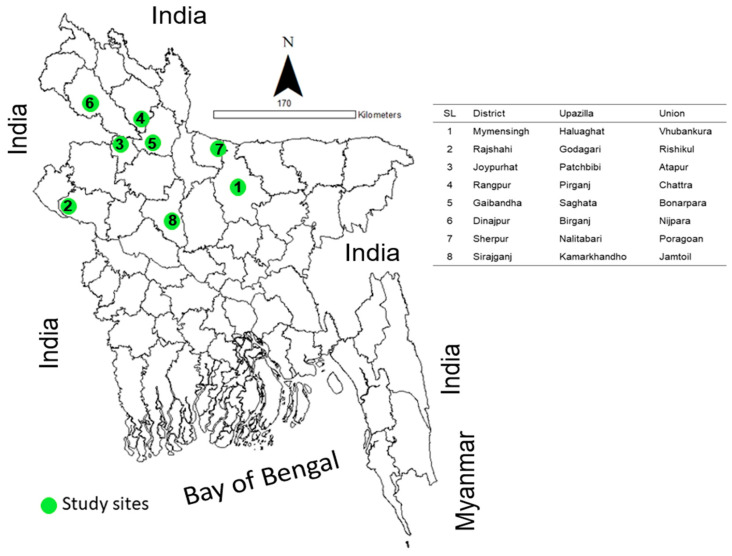
A geographical map of Bangladesh showing the locations of the study areas.

The study was conducted in eight districts and eight sub-districts (Upazilla), including our proposed anthrax free model area of Bangladesh. In each sub-district, we selected one union (lowest administrative unit of local government). Nine locations were selected in each union for the mass vaccination program. These locations were carefully selected with the consideration of the farmers’ convenience. Three focus group discussions were conducted with the selected community people in each union, and one validation workshop was done in each Upazilla headquarter. Since our study was conducted in the northern part of Bangladesh and each region had minor variations in literacy, awareness level, socio-economic status, population density, as well as cattle number, therefore these variations were overlooked in the present study. The geographical location of the study sites is shown on the map in Figure 1.

### 2.3. Anthrax Vaccine Used for Mass Vaccination

The anthrax vaccine used for our vaccination campaign was collected from the Livestock Research Institue (LRI), Mohakhali, Dhaka, Bangladesh, through Upazilla and district office of the Department of Livestock Services. The vaccine was contained in a 100 mL bottle, and each ml contained approximately 1 × 10^7^ attenuated live spores of *Bacillus anthracis* (*B. anthracis*). According to the manufacturer’s (LRI, Dhaka, Bangladesh) recommendation, the dose to be used for cattle was 1 mL administered as subcutaneous injection. All cattle over six months of age were covered under a mass vaccination program. Pregnant cows of age more than six months were not vaccinated.

### 2.4. Organization of Campaigns and Vaccinated Campaign for Cattle

The study research team, along with the Upazilla livestock office of the respective sub-district, jointly implemented the cattle vaccination campaign from 2017 through 2018. Mennonite Central Committee Bangladesh (an international, voluntary and nonprofit organization) supported the necessary arrangements for the campaigns like community announcements, informed potential local leaders, religious leaders, ward chowkidar and elite persons. Before the campaign, a cattle census was performed in each catchment area to record the total cattle population, including age and pregnancy status, to calculate the number of cattle for anthrax vaccination coverage. Before vaccination campaigns, trained community-based facilitators of Mennonite Central Committee Bangladesh followed standardized instructions from the study research team to inform the majority of cattle-owning households about the place, date, and time of the vaccination spots by personal contact, peer contact, and contact with religious and local leaders and elites.

### 2.5. Livestock Census

A census was taken on the livestock after each vaccination campaign. Before performing the census, all the concerned workers were trained on the data collection methodology. The staff counted the total cattle population, the number of pregnant cows of age more than 6 months, the number of calves aged below 6 months and the number of cattle that had been vaccinated by door-to-door visit.

### 2.6. Focus Group Discussion

Focus group discussions (FGDs) were used to collect qualitative information about the existing vaccination techniques, explore the possible barriers, and look for possible ways to overcome these barriers. Three FGDs were conducted in each Upazilla considering time and resource limitations. Multistage purposeful sampling and maximum variation sampling were used to choose the participants. Each FGD had 7–11 people that were randomly selected. Before starting the session, a facilitator introduced the participants to the purpose of the session and ensured all the findings would only be used for research purposes. Keeping the respect for each participant, the facilitator asked the same selective questions. Sessions typically lasted one to two hours. All the findings were documented in writing and keynotes were also repeated as the summary of the discussion for further confirmation.

### 2.7. Key Informer Interview

Key informer interviews (KIIs) were conducted in every Upazilla with staff involved in the livestock mass vaccination program. Veterinary field assistants, locally trained vaccinators, veterinary surgeons, and Upazilla livestock officers were interviewed. The purpose of the key informer interviews (KIIs) was to collect information about the context, culture, limitation, scope, and anthropological pattern in the respective community. All information was documented for validation of the FGD findings.

### 2.8. Validation of FGD Findings

One validation workshop was held in each Upazilla with the key stakeholders who had been involved in the earlier process. Participants included a representative from the university, the Department of Livestock Services, community vaccinators, and local community elites. All the findings of the census, FGDs, KIIs were represented in the validation workshop. The main goal of the validation workshop was to create a sharing platform to review the findings of the FGD and provided another scope to add more information if anything was not mentioned. The participants asked about the method and findings of the present study and were given further clarification. Finally, based on their consensus, the FGD findings were taken for publication and completed the data processing. In the validation workshop, the added findings were also recorded.

### 2.9. Quantitative and Qualitative Data Analysis

All data of the censuses, FGD, and KII were transferred to excel worksheets, and descriptive statistics were carried out using Microsoft Excel^®^ tools and the results were expressed as frequencies and proportions. Categorical responses were presented as proportions and their associations were determined using the *Chi*-square tests.

## 3. Results

### 3.1. Livestock Anthrax Vaccination Rate

**Figure 2:**
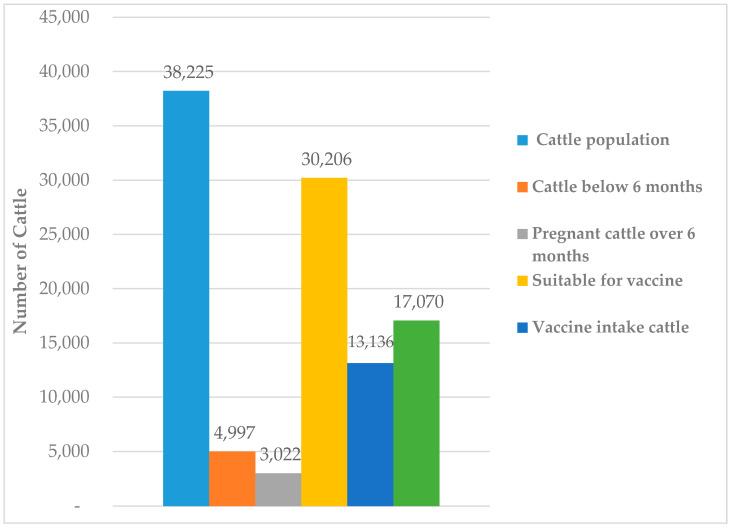
The cumulative Livestock census and coverage of vaccine intake during vaccinations in the present study.

A total of 13,136 cattle were vaccinated in 72 vaccination campaigns in 8 different study areas. In those catchment areas, there were 38,225 cattle; among them, 4997 were under six months of age, and 3022 were pregnant cows over six months. The availability of cattle for vaccination was 30,206. The vaccine was administered to 13,136 cattle, thus results in 44% coverage. The remaining 66% of vaccination-available cattle remained unvaccinated (Figure 2). The breakdowns of sampling spots, numbers of cattle in total, under six months, pregnant cows more than six months, cattle available for vaccination, unvaccinated cattle, and vaccine coverage of the different districts was shown in Table 1.

### 3.2. Barriers to Vaccine Uptake

**Figure 3:**
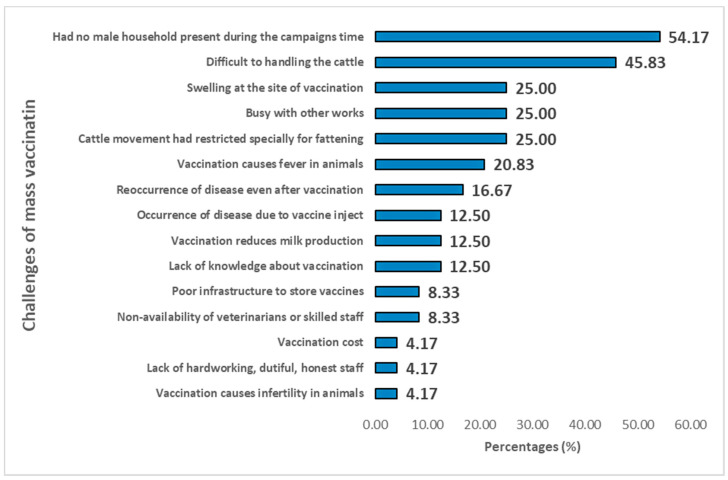
The constraints for mass vaccinations identified in the FGDs in the present study.

It was found that 15 constraints against anthrax vaccine uptake in livestock were identified from 24 FGDs conducted in the study areas. During the study, one of the significant hurdles that surfaced up is that usually no male household is present at home, due to their work, at the time of the vaccination campaign. Another prominent constraint was the difficulties faced when handling the cattle. These constraints were identified by 54.17% and 45.83% of the FGDs participants respectively. Other major constraints were identified in each 25% of the FGDs such as restricted cattle movement, especially for fattening (many farmers believe that fattening animal should not be walked), being busy with other work, and the swelling at the site of vaccination. Four constraints were identified in 10–25% of the FGDs and included fever caused by vaccination in animal (20.83%), the occurrence of the disease despite vaccination (16.66%), reduced milk production due to vaccination (12.5%), and the lack of farmer’s knowledge about vaccination (12.5%). Further constraints identified in less than 10% of the FGDs were infertility in animals caused by vaccination, the absence of veterinarians or skilled staff, poor infrastructure to store vaccines, the lack of hardworking, honest staff, and the vaccination costs (Figure 3).

### 3.3. Recommendations to Maximize Vaccine Uptake

During FGDs, a total of 17 recommendations were identified to maximize anthrax vaccine uptake. The most prominent recommendations identified were individual home delivery service (identified by 58.33% of the FGD participants), arranging anthrax vaccination campaigns every six months (54.17%), increasing the number of veterinarians, vaccinators, or skilled staff (45.83%), proper storage and following the expiry date of anthrax vaccines in the Upazilla livestock office (33.33%), informing farmers about the vaccines and investigating apparent vaccination failure (25%) and reasons of the reoccurrence of disease even after vaccination (25%). The recommendations identified by the FGD participants, as well as information on FGDs are shown in Table 2.

### 3.4. Validation of the FGD Findings

Participants of KIIs and validation workshops agreed to all of the research findings regarding the constraining of vaccine uptake and the recommendations. Additionally participants in the validation workshop added that the vaccine should be more effective to preventive anthrax disease (22.9%), shortage of manpower in the livestock sector (22.5%), poor extension services (21.2%), lack of knowledge and poor attitude of the cattle farmers (10.5%), lack of coordination between demand and vaccine supply (11.2%), and livestock owners were not willing to pay the vaccination cost (10.5%). (Details are illustrated in Table 3).

## 4. Discussion

The livestock population in Bangladesh is scattered all over the country, but the density is not similar. Smallholder cattle farmers are located mostly in Pabna, Sirajganj, Kushtia, Khulna, Rangpur, Dinajpur, Naogaon, and Chittagong districts [24,25]. It was also observed in our vaccination campaign records that the cattle number was different from one place to another. The livestock vaccination campaign against anthrax demonstrated that we had only 44% coverage of the total cattle population, and thus 66% of cattle population remained unvaccinated. Poor vaccination coverage rates of anthrax vaccine in Bangladesh were previously reported and it was significantly poor and not more than 25–40% of the total cattle population [12,13,26]. This poor coverage of anthrax vaccination uptake was also observed in most of the low-income countries where livestock is reared by households [27,28,29]. In 1974, the World Health Organization (WHO) introduced the Expansion Program on Immunization (EPI) that was implemented all over the world [30]. In this program, there has been tremendous progress in vaccine uptake in humans [31]. Through this program, immunization service and coverage markedly improved, and many countries of the world ensured full vaccination coverage for certain diseases [32]. As a result, several devastating infectious diseases such as diphtheria, whooping cough, tetanus, poliomyelitis, and measles are currently under control [32]. However, there has been no similar initiative in the livestock sector. Furthermore, the extension work is done to increase the awareness level, and vaccination uptake in livestock has mostly been aimed at small and large farm levels [33]. Livestock rearing at the household level has rarely been targeted. At the farm level, livestock vaccine acceptance increased five times in the last ten years compared to 1.5 times at the household level, thus increasing the gap between them [33,34,35]. Farmers sell more adult males than females or young animals, leaving a herd structure with a relative predominance of female, pregnant cows and calves. In our study, this was reflected as 20% of cattle were unable to be vaccinated. The relatively large part of herds being unable to be vaccinated was also a finding in our KIIs and validation workshops. Participants of validation workshops reported that rural households preferred to treat not to prevent the disease. Therefore, farmers did not want to vaccinate their cattle in a healthy condition. Sometimes, they confused treatment with vaccination, which was also observed in other South Asian countries such as India, Nepal, and Pakistan [36,37,38,39]. In the present stusy, approximately 70% of the livestock was reared by small scale farmers and landless labors, who control less than 30% of the land area and farmers had their livestock vaccinated only when a veterinarian or para- veterinarian visited their village under government schemes such as FMD-Control Program, Brucellosis-Control Program, and others [40,41,42]. Poor coverage of livestock anthrax vaccination and ineffective vaccination in some livestock animals contributed to outbreaks of the disease [12,13,43]. The improvement of anthrax vaccination coverage among livestock is also one of the most critical control strategies to control anthrax in Bangladesh. Barriers to vaccine uptake reported from our study were the absence of male family member at the time of the vaccination program, difficulty to handle the cattle, restriction of cattle movement especially for fattening, being busy with other works, the lack of knowledge about vaccination, reduction of milk production due to vaccination, infertility in cows due to vaccination, swelling at the site of vaccination, fever in animals due to vaccination, reoccurrence of the disease after vaccination, shortage of veterinarian, poor infrastructure and vaccination cost. The National Livestock Extension Policy has been constructed to strategically address the challenges and constraints prevailing in livestock production systems [24]. Among their findings, inappropriate organizational setup with inadequate extension manpower and inadequate animal health care services were similar to our findings. The current study has demonstrated that the unavailability of male person being present at the vaccination campaign who help bring the animal to the vaccination spot is considered one of the greatest barriers to vaccine uptake of livestock. National data also showed that 79.9% of households are small-scale farmers holding 0.05–2.49 acres of land, whilst 10.2% of households have no land [25]. The dynamics of rural male labor shift from farming to non-farming activities such as trade, business, and services. Males usually work outside of their houses, and 90% of rural females are responsible for rearing livestock and poultry. However, the participation of females was relatively low in activities such as bringing the animals to the veterinary hospital for treatment, sale of animals, breeding of animals, fodder cultivation, and construction of animal shed. A large number of cattle are slaughtered annually for religious purposes and domestic consumption in Bangladesh. Therefore beef fattening has become popular in rural areas, and cattle movement has been restricted, especially for fattening programs, which was also observed as a proposed vaccination constraint in our study.

In Bangladesh, the Australia-origin Sterne F-34 vaccine strain of *B. anthracis* is used to prevent anthrax in animals. It has some reported side effects such as the injection site may itch, followed by swelling and discoloration, and sometimes fever or reduced milk production for a few days. A study on Sterne F-34 strain vaccine in cattle and goat found similar side effects after vaccination [20]. The government of Bangladesh sets a subsidized price for most of the livestock anthrax vaccine, approximately US $0.59 (BDT 50), for a vial of 100 doses. However, sometimes, the vaccinator unofficially charged the farmers US $0.12 (BDT 10) extra for a single dose of vaccine to cover their additional expenses [13]. On the other hand, most livestock offices had some volunteers called local service providers or community animal vaccinators who get no or minimal honorarium. Usually, these workers conducted livestock vaccination campaigns, and sometimes they demand additional money for their service from the farmers. Sometimes, the Upazilla livestock department compensates vaccine costs via other development projects or collecting donations from others, which creates confusion among farmers. This flexibility created another confusion. The farmers did not want to pay because they believed a government-supplied vaccine should be free of charge. The Upazilla Livestock officers also explained this issue to farmers that the amount did not cover their cost to travel to distant villages, particularly in remote areas, and thus they were not willing to go there. The officers suggested the majority of these problems could be solved by increased manpower in the upazilla hospitals and by increased transport facilities. These measures would be of significant importance for improved vaccination coverage in Bangladesh. Other researchers have reported various reasons for poor vaccination coverage, such as the acute shortage of the anthrax vaccine and a shortage of veterinary facilities, including manpower [12,13,18]. Anthrax vaccination was strongly linked to social and cultural rather than economic drivers [14,15,16,17]. From the present study, stakeholder recommendations included individual home delivery service, anthrax vaccination campaigns every six months, information about anthrax disease and vaccination benefits, and the improvement of vaccine quality to reduce adverse effects. It is also recommended that the Livestock Research Institute (LRI) should take the initiative to improve the anthrax vaccine quality. The findings of the present study demonstrates that vaccine uptake is a complex multifactorial issue, where responsibility is shared by farmers, veterinary departments, district, local, and national governments, and vaccine producers.

## 5. Conclusions

Human anthrax is mainly zoonotic, and thus the control of animal disease is the best mitigation strategy. This study demonstrates that anthrax vaccine uptake was only 44% in the study area in Bangladesh. Major identified constraints for vaccination include unavailability of male being present in household during the time of vaccination campaigns, difficulties in handling the cattle, practice of restricted cattle movement, especially for fattening, cattle owners being busy with other works, swelling at the site of vaccination, fever in the animal caused by vaccination, lack of knowledge about vaccination, reduction of milk production due to vaccination, and poor vaccine effect. Identified recommendations to improve vaccine uptake include individual home delivery service and to arrange anthrax vaccination campaigns every six months.

## Figures and Tables

**Table 1:** The Livestock population and average vaccination intake in different study locations.

District	Spot	Cattle Population (MEAN ± SE)	Under 6 Months (MEAN ± SE)	Pregnant over 6 Months (MEAN ± SE)	Available Cattle for Vaccination (MEAN ± SE)	Actual Vaccinated (MEAN ± SE)	Unvaccinated (MEAN ± SE)	Percentage (%)
Mymensingh	9	601 ± 68	74 ± 12	59 ± 9	468 ± 60	229 ± 31	239 ± 39	49
Rajshahi	9	542 ± 47	45 ± 6	33 ± 3	464 ± 48	205 ± 14	259 ± 41	47
Joypurhat	9	379 ± 53	60 ± 4	35 ± 5	284 ± 47	120 ± 17	164 ± 31	44
Rangpur	9	466 ± 44	72 ± 6	38 ± 3	356 ± 39	151 ± 14	205 ± 41	42
Gaibandha	9	633 ± 43	71 ± 8	45 ± 7	517 ± 49	206 ± 24	311 ± 33	40
Dinajpur	9	483 ± 34	75 ± 13	44 ± 3	364 ± 41	151 ± 15	213 ± 34	41
Sherpur	9	528 ± 59	88 ± 5	41 ± 3	399 ± 53	192 ± 25	207 ± 31	48
Sirajganj	9	613 ± 35	67 ± 6	37 ± 3	509 ± 32	203 ± 18	306 ± 28	39

**Table 2:** Key recommendations for maximizing anthrax vaccine uptake form the Focus Group Discussions in the present study.

Location	1	2	3	4	5	6	7	8	Total	Percentage (%)
Information on FGDs	-	-	-	-	-	-	-	-	-	-
Number of FGDs	3	3	3	3	3	3	3	3	24	-
Total number of participants	24	27	21	33	33	24	24	30	216	-
Recommendations identified	Number of participants identifying recommendation									-
Individual Home delivery service	18	18	9	27	18	9	18	9	126	58.33
Arrange anthrax vaccination campaigns every 6 months	9	18	9	9	27	9	9	27	117	54.16
Inform farmers about the anthrax disease and how to control it	9	-	9	-	-	-	-	9	27	12.5
Inform farmers about the benefits of vaccination	9	-	-	-	18	9	9	9	54	25
Carry out additional research to study potential causes of reduction in milk production post-vaccination, and find solutions to the problem	-	9	-	-	-	9	-	9	27	12.5
Investigate whether or not vaccination causes infertility in animals	-	-	-	-	-	-	9	-	9	4.16
Investigate why swelling at the site of vaccination occurs and how it can be controlled	-	9	-	-	-	9	-	9	27	12.5
Study the causes of fever in animals after vaccination	-	-	9	-	-	-	9	-	18	8.33
Investigate apparent vaccination failure, leading to the reoccurrence of disease even after vaccination	9	9	9	9	9	9	-	-	54	25
Increase the number of veterinarians, vaccinators or skilled staff	18	9	9	18	9	9	18	9	99	45.83
Establish a reliable cold chain to store and transport vaccines	9	9	-	-	-	-	9	9	36	16.66
Properly store and follow the expiry date of vaccines in Upazilla livestock office	9	9	9	9	9	9	9	9	72	33.33
Carry out research to produce vaccines that can be stored at room temperature (thermostable/thermotolerant).	9	9	9	-	-	-	-	-	27	12.5
Free of charge vaccinations	-	-	-	9	-	-	-	-	9	4.16
Increase collaboration of Livestock Local vaccinators with veterinarians and Livestock department	9	-	-	-	-	-	-	9	18	8.33
Impose fine or punishment if failing to vaccinate	-	9	-	-	-	9	-	-	18	8.33
Involve law enforcing agencies and local administration	-	-	-	9	-	-	9	-	18	8.33

Location: 1 = Mymensingh; 2 = Rajshahi; 3 = Joypurhat; 4 = Rangpur; 5 = Gaibandha; 6 = Dinajpur; 7 = Sherpur; 8 = Sirajganj.

**Table 3:** Additional recommendation for maximizing vaccine uptake form Validation Workshop.

Location	1	2	3	4	5	6	7	8	Total	Percentage (%)
Information on Validation Workshop	-	-	-	-	-	-	-	-	-	-
Number of validation workshop	1	1	1	1	1	1	1	1	8	-
Total number of participants	57	53	52	54	48	47	51	39	401	-
Recommendations identified	Number of participants identifying recommendation									-
Vaccine should be more effective	12	20	8	13	7	6	18	8	92	22.9
Lack of knowledge and poor attitude by cattle farmers	6	6	7	8	5	4	7	3	46	11.4
Farmer are not willing to pay the vaccination cost	6	5	6	8	10	4	1	2	42	10.5
Lack of Coordination between demand and vaccine supply	12	4	5	3	-	7	10	5	46	11.2
Shortage of Manpower in Livestock sector	13	10	14	7	9	8	23	6	90	22.5
Poor extension services	11	18	6	18	11	13	2	6	85	21.2

Location: 1 = Mymensingh; 2 = Rajshahi; 3 = Joypurhat; 4 = Rangpur; 5 = Gaibandha; 6 = Dinajpur; 7 = Sherpur; 8 = Sirajganj.
